# Supplementary material for: Cdk5 mediates rotational force-induced brain injury
Source: Sci Rep. 2023 Feb 28;13:3394. doi: 10.1038/s41598-023-29322-4 (PMC9974974; doi:10.1038/s41598-023-29322-4)
Supplement: Supplementary file 1 — Supplementary Information. [file 41598_2023_29322_MOESM1_ESM.pdf]

Supplemental Figure 1. Umfress, *et al.*

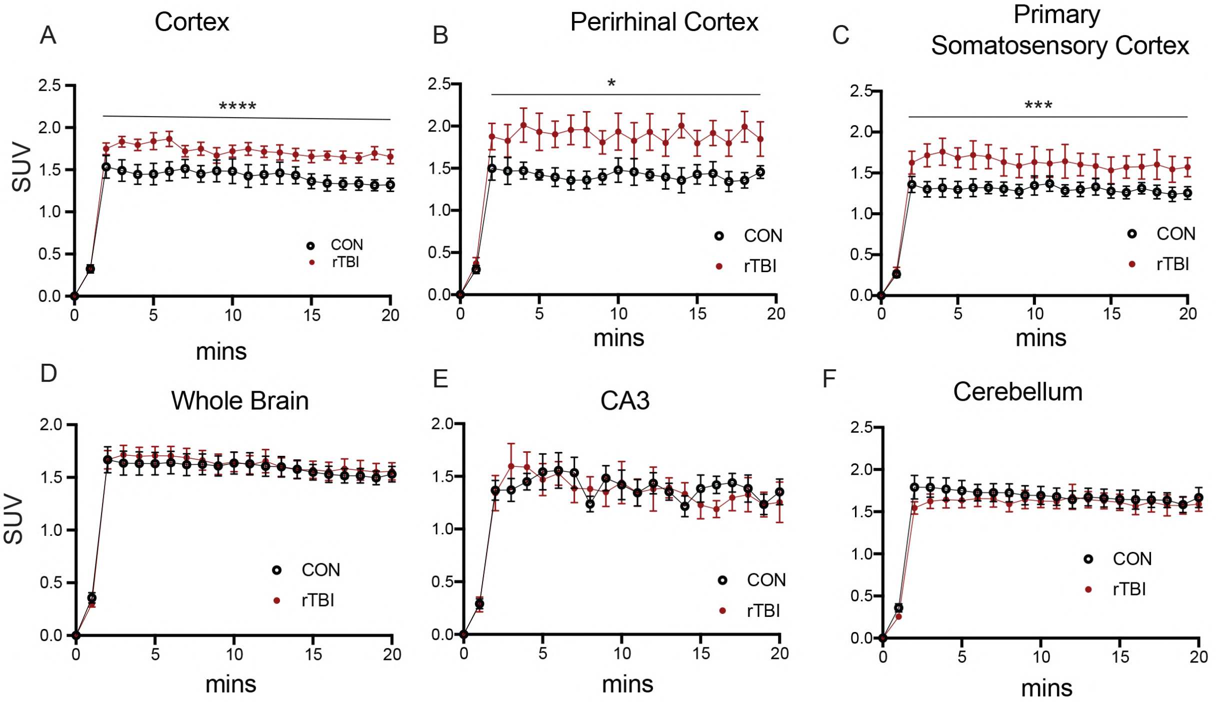

Supplemental Figure 1. PET/CT analysis of diffuse TSPO uptake into the brain Standard uptake values in A. Cortex (Time: $F(20,200) = 197.0$, $p < 0.0001$; Treatment: $F(1, 10) = 5.580$, $p = 0.0398$; Interaction: $F(20,200) = 3.063$, $P < 0.0001$) two-way-RM ANOVA B. Perirhinal cortex (Time: $F(20,200) = 61.32$, $p= 0.0130$; Treatment: $F(1,10) = 6.020$, $p = 0.0341$; Interaction: $F(20,200) = 1.917$, $p = 0.0130$) C. Primary somatosensory cortex (Time: $F(20, 200) = 132.4$, $p < 0.0001$; Treatment: $F(1,10) = 3.418$, $p = 0.0942$; Interaction: $F(20,200) = 2.374$, $p = 0.0013$). D. Whole brain (Time: $F(20,200) = 402.6$, $p<0.0001$; Treatment: $F(1,10) = 0.06724$, $p = 0.8007$; Interaction $F(20,200) = 0.8649$ E. CA3 hippocampal layer (Time: $F(20,200) = 47.91$ $p < 0.0001$; Treatment: $F(1,10) = 0.02460$, $p=0.8785$; Interaction $F(20,200) =1.030$, $p = 0.4283$). F. Cerebellum (Time: $F(20,200) = 277.4$ $p < 0.0001$; Treatment $F(1,10) = 0.3043$ $p = 0.5933$; Interaction $F(20,200) = 1.255$, $p = 0.2138$. All data are means ± SEM, *p < 0.05, *< 0.01, ***< 0.001. All statistical analysis consisted of two way-RM ANOVA.

Supplemental Figure 2. Umfress, *et al.*

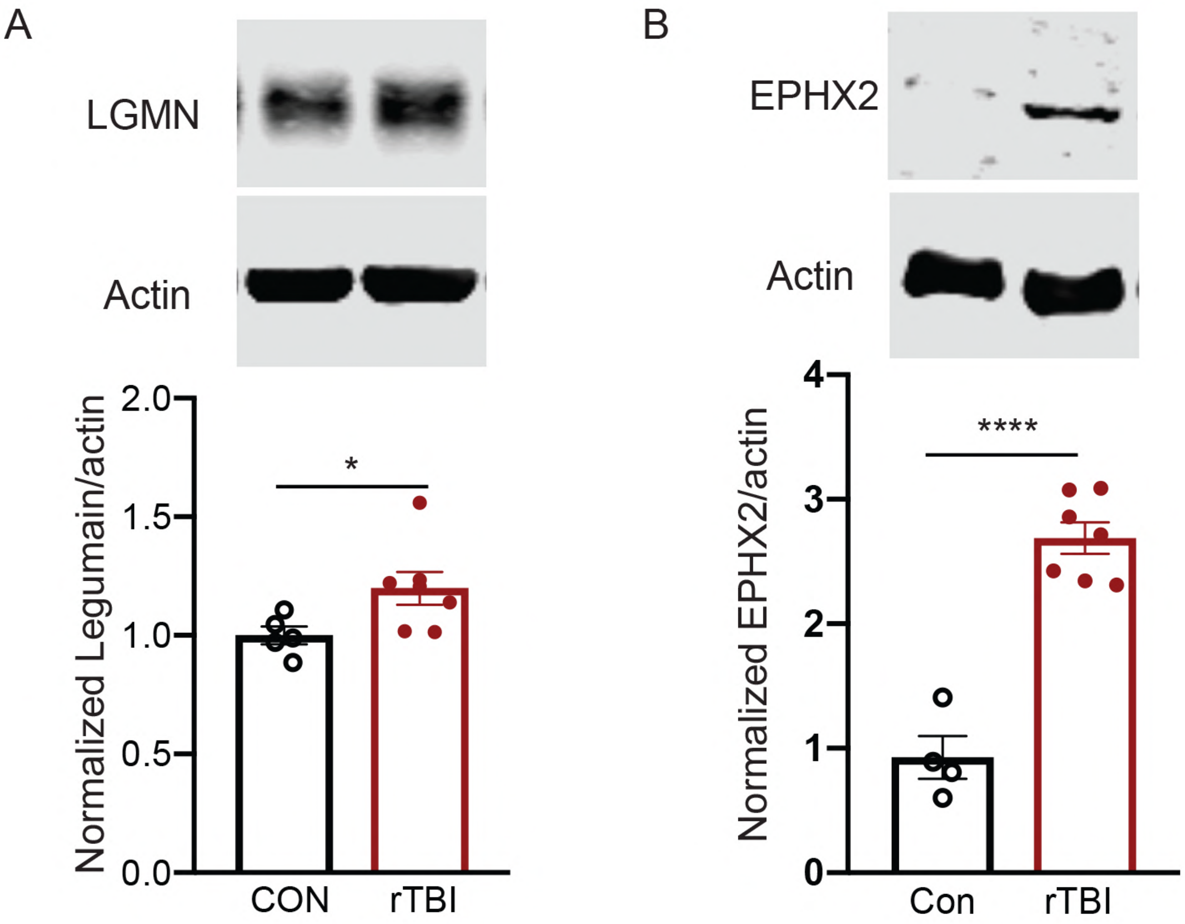

Supplemental Figure 2. Validation of proteomic changes. A. Quantitative immunoblot for protein expression ratio of LGMN/ actin, p = 0.0494 Student's *t*-test. B. Quantitative immunoblot for protein expression ratio of EPHX2/ actin, p < 0.0001. Data are means ± SEM, *p < 0.05, ****< 0.0001. Full uncropped blots available in Supplementary Figure 6.

Supplemental Figure 3. Umfress, *et al.*

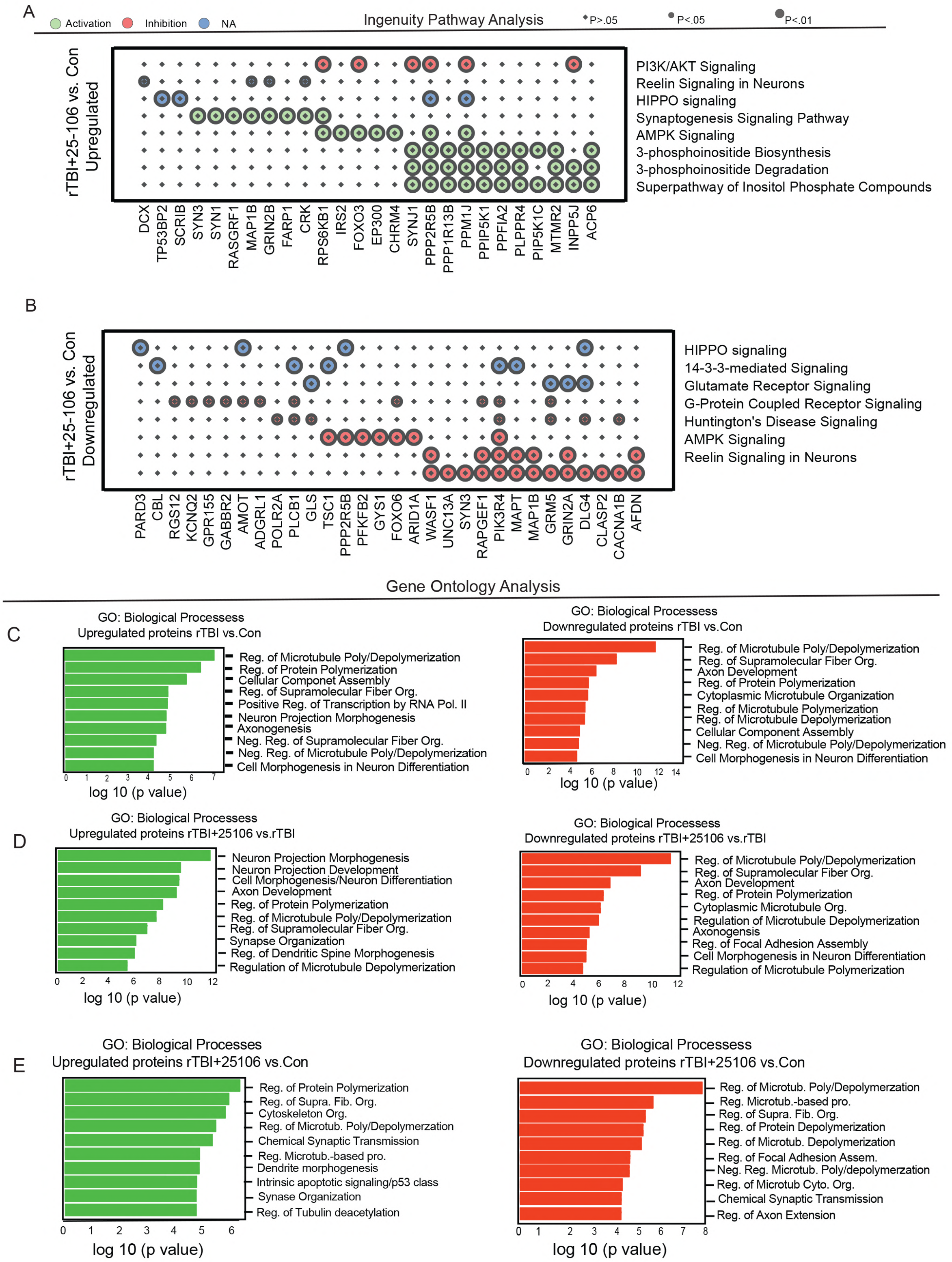

Supplemental Figure 3. Pathway and ontological analysis of rTBI+25-106 versus Con rats. A. Dot-plot display of Ingenuity Pathway Analysis of phospho-proteins and canonical pathways upregulated FC≥1.3 by rTBI + 25106:Con (Top) and B. downregulated with a FC ≤ -1.3 by rTBI

+ 25-106:Con (bottom). C. Gene ontology of phosphoproteins biological processes upregulated FC≥ 1.3 (left, green) or downregulated FC ≤ -1.3 (right, red) in rTBI + 25-106:Con. C. Gene ontology of phosphoproteins biological processes upregulated FC≥ 1.3 (left, green) or downregulated FC ≤ -1.3 (right, red) in rTBI + 25-106:TBI. D. Gene ontology of phosphoproteins biological processes upregulated FC≥ 1.3 (left, green) or downregulated FC ≤ -1.3 (right, red) in rTBI + 25-106:Con. E. Gene ontology of phosphoproteins biological processes upregulated FC≥ 1.3 (left, green) or downregulated FC ≤ -1.3 (right, red) in rTBI + 25-106:Con.

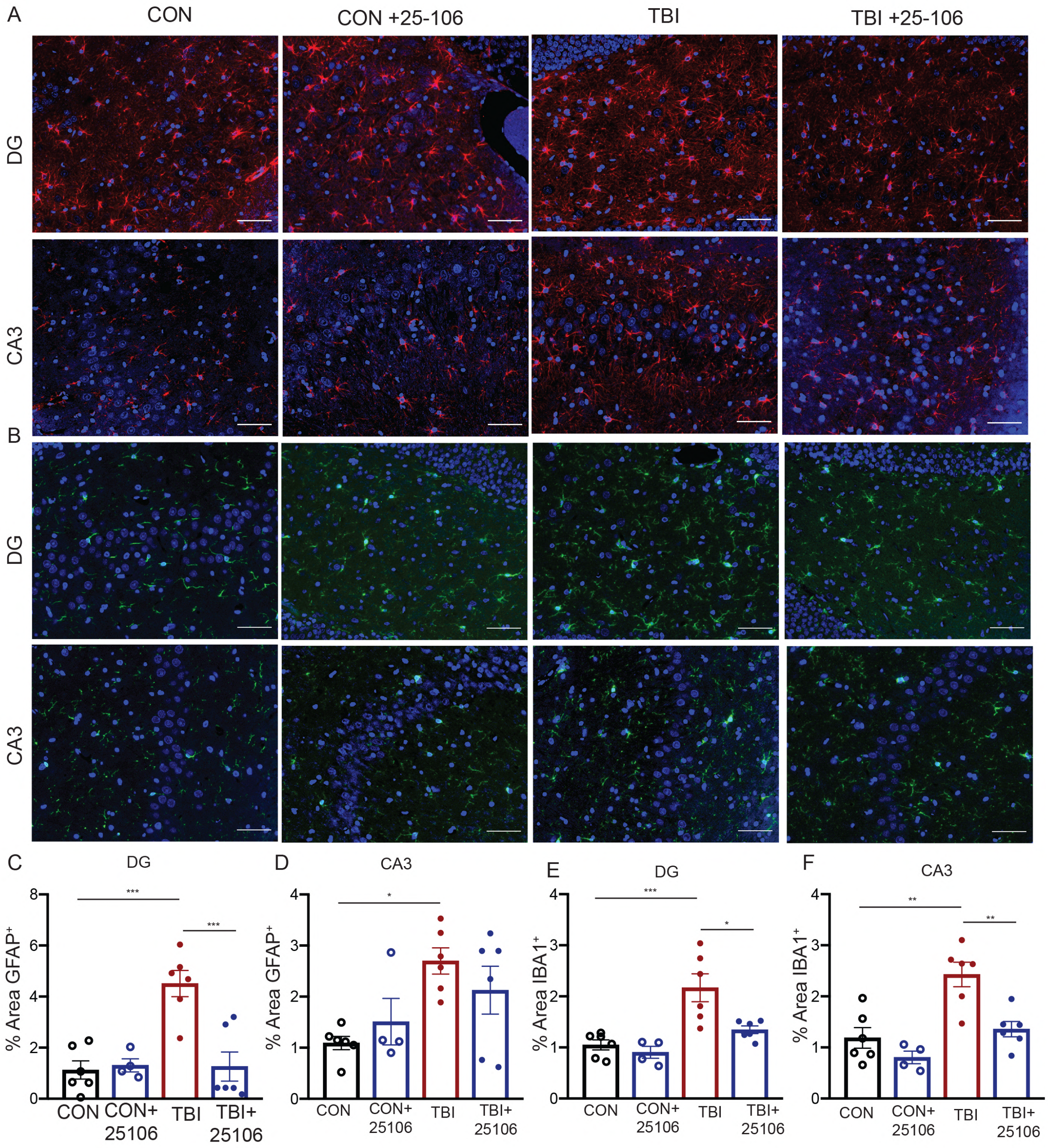

Supplemental Figure 4. Cdk5 inhibition provides *in vivo* neuroprotection. Immunohistochemical staining and quantitation of GFAP+ astrocytes within the dentate gyrus (DG. A, C) $F(3,18) = 12.74$, $p = 0.0001$ ANOVA and CA3 (A, D) $F(3,18) = 4.494$, $p = 0.0160$ ANOVA. Immunohistochemical staining and quantitation of Iba1+ microglia within the DG (B, E), $F(3,18) = 11.11$, $p = 0.0002$ ANOVA and CA3 (B, F), $F(3,18) = 12.33$, $p =0.0001$ ANOVA All data are means ± SEM, $p < 0.05$, $^{**}<0.01$. Scale bars = 100 μm.

Supplemetary Figure 5. Umfress, *et al*.

Figure 5A

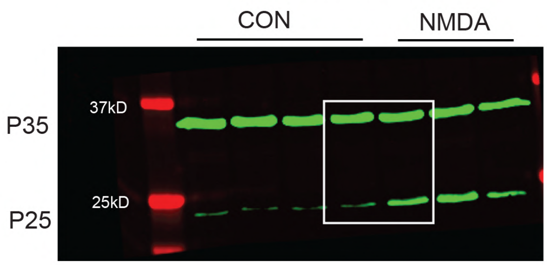

Figure 5B

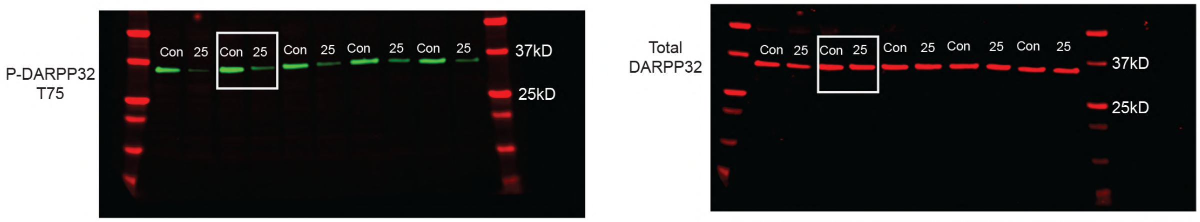

Figure 5D

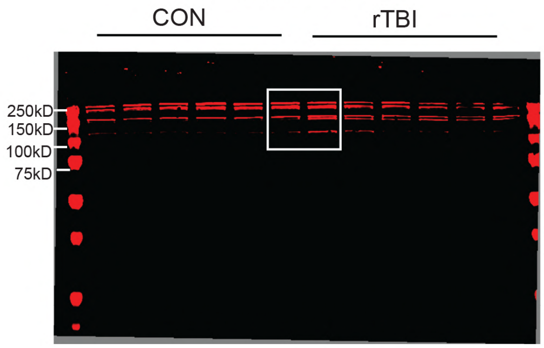

Figure 5E

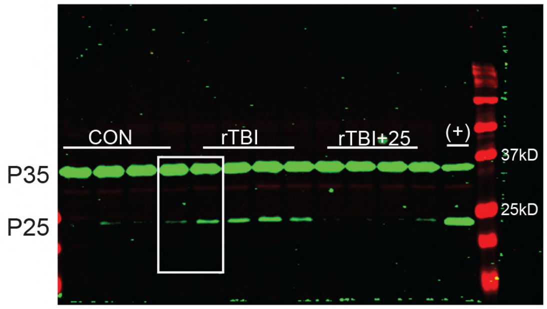

Figure 5F

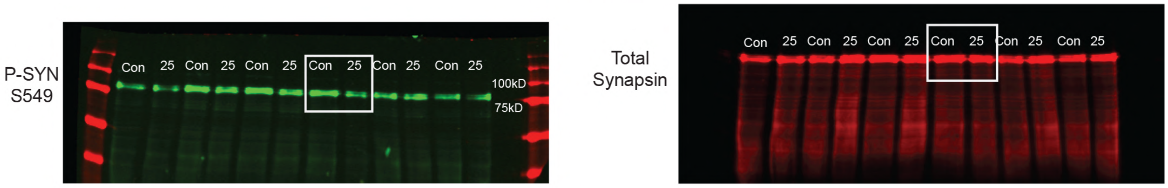

Supplemetal Figure 2A

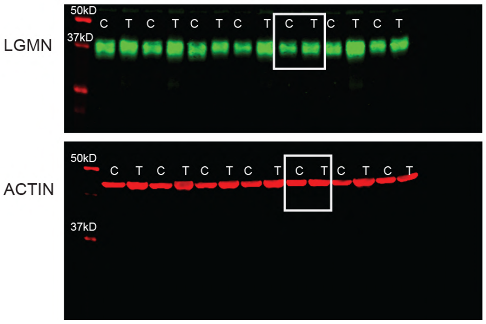

Supplemetal Figure 2B

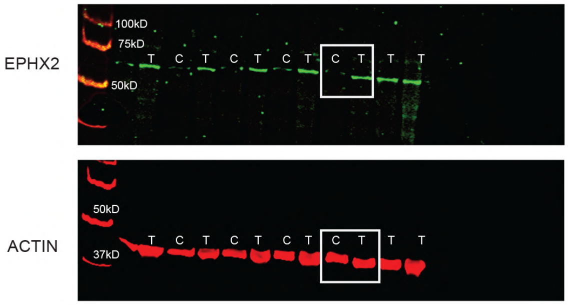

Supplemental Figure 5. Full blots used in the main text. Entire immunoblots are shown for each figure as indicted with appropriate molecular weight markers and labels. Abbreviations: Con = control, NMDA = N-methyl-D-aspartate, 25 = 25-106, C = control, T = TBI. Cropped blots used in main text outlined in white boxes.
